# Advancing stochastic 3-SAT solvers by dissipating oversatisfied constraints

**DOI:** 10.1073/pnas.2517297122

**Published:** 2025-11-14

**Authors:** Joachim Schwardt, Jan Carl Budich

**Affiliations:** ^a^Condensed Matter Division, Max Planck Institute for the Physics of Complex Systems, Dresden 01187, Germany; ^b^Institute of Theoretical Physics, Technische Universität Dresden and Würzburg-Dresden Cluster of Excellence ct.qmat, Dresden 01062, Germany

**Keywords:** Boolean satisfiability, stochastic local search algorithms, NP-complete problems

## Abstract

We introduce and benchmark a stochastic local search heuristic for the Nondeterministic Polynomial Time (NP) complete satisfiability problem 3-SAT that drastically outperforms existing solvers in the notoriously difficult realm of critically hard instances. Our construction is based on the crucial observation that well established previous approaches such as WalkSAT are prone to get stuck in local minima that are distinguished from true solutions by a larger number of oversatisfied combinatorial constraints. To address this issue, the proposed algorithm dissipates oversatisfied constraints (DOC), hence coined DOCSAT, i.e. reduces their unfavorable abundance so as to render them critical. We analyze and benchmark our algorithm on a randomly generated sample of hard but satisfiable 3-SAT instances with varying problem sizes up to *N* = 15,000. Quite remarkably, we find that DOCSAT outperforms both WalkSAT and other well known algorithms including the complete solver Kissat, even when comparing its ability to solve the hardest quintile of the sample to the average performance of its competitors. The essence of DOCSAT may be seen as a way of harnessing statistical structure beyond the primary cost function of a combinatorial problem to avoid or escape local minima traps in stochastic local search, which opens avenues for generalization to other optimization problems.

## Significance

Hard decision problems, in computational complexity theory known as NP-complete, are of universal importance. From a conceptual perspective, an efficient solution to one such complete problem is tantamount to solving any other in the wide class of efficiently verifiable (NP) problems. Moreover, paradigmatic examples such as the Boolean satisfiability problem 3-SAT have ubiquitous applications ranging from circuit design to logistics. In this paper, we introduce the heuristic algorithm DOCSAT that drastically outperforms previous methods to tackle 3-SAT in the notoriously difficult regime of barely satisfiable randomly generated instances. There, our DOCSAT solver pushes the feasible problem sizes by about an order of magnitude, quite remarkably so in light of the asymptotically exponential cost reflecting the NP-completeness of the considered problem.

**Fig. 1.**
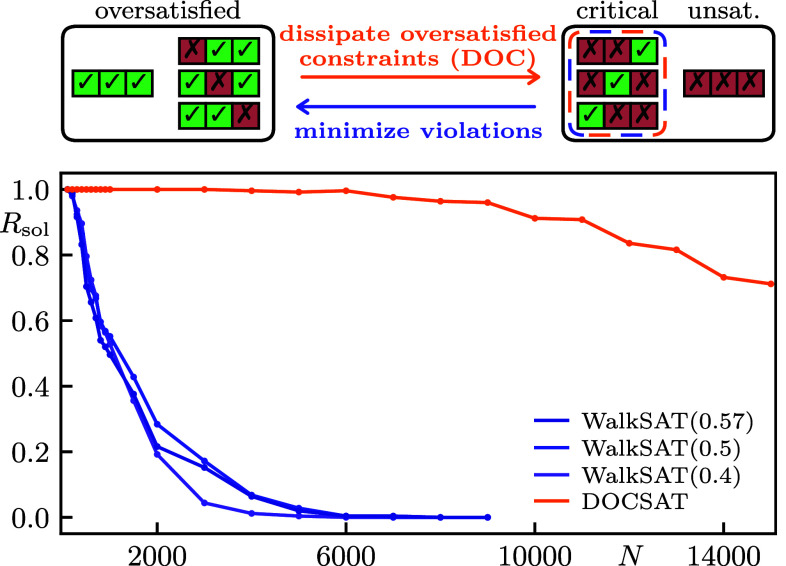
*Upper* panel: Illustration of two competing processes based on the 8 logical types of 3-SAT constraints. Minimizing violations, i.e. unsatisfied constraints, typically leads to excessive oversatisfied ones, which is counteracted by DOC. The desirable critical clauses are highlighted as a compromise of both processes. *Lower* panel: Fraction $R_{\text{sol}}$ of solved 3-SAT instances as a function of the number of variables N for WalkSAT (30) and DOCSAT (cf. *Algorithm* 1). At every N, 250 instances at critical clause density $\alpha =\alpha_{\text{crit}}=4.27$ are generated using the Weigt-protocol (31). The solvers are run $10^{3}$ times with 300N iterations per trial. The various walk probabilities $P_{\text{walk}}$ for WalkSAT are displayed in the legend, while $P_{\text{walk}}=0.4$ and $r_{\text{doc}}=0.15$ are fixed for DOCSAT.

**Fig. 2.**
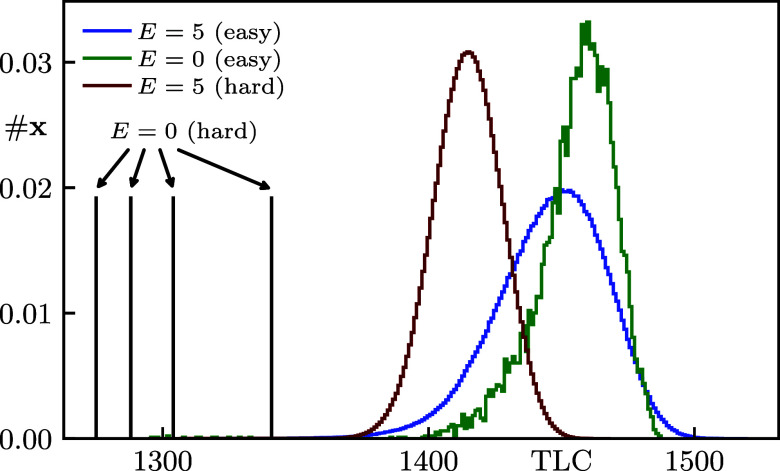
Density of assignments #x at a fixed energy E (Eq. **1**) as a function of TLC (Eq. **2**) for $10^{4}$ runs of WalkSAT with $10^{4}$ flips per run and $P_{\text{walk}}=0.5$. Out of 250 instances with N=200, those with success probability P>90% (easy) and P<1% (hard) are compared. The black vertical stilts correspond to individual solutions of four hard instances. For easy instances, differences in TLC between the low-energy states (E=5) and solutions (E=0) are small; in both cases, the average TLC is roughly 1,450. For hard instances, the distribution of low-energy states (E=5) is instead largely disjoint in TLC from the solutions (E=0), i.e. there is a large difference in the average TLC. This indicates that the regime of low E and large TLC constitutes a vast landscape of local minima for WalkSAT, from which solutions of hard instances are difficult to reach by local search.

**Fig. 3.**
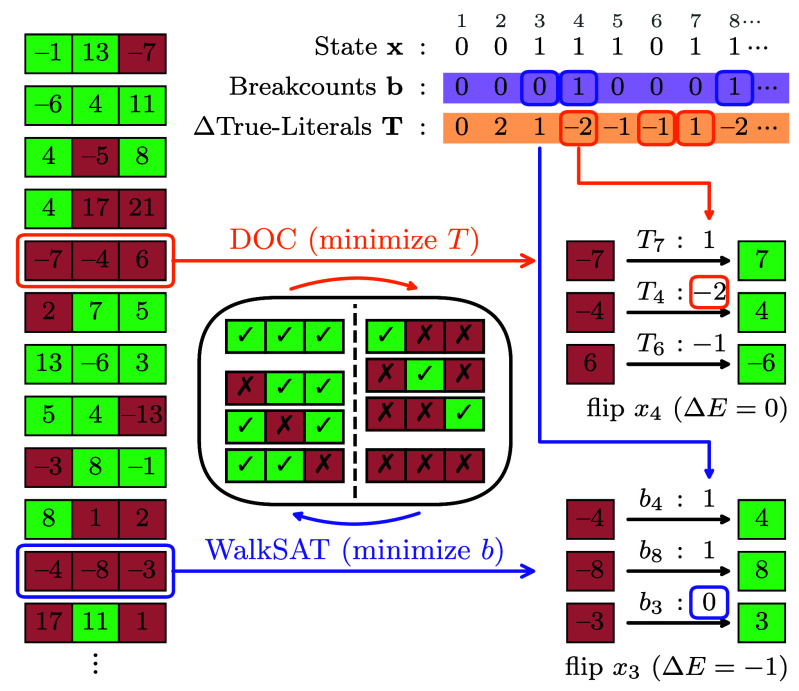
Illustration of the variable selection heuristic in DOCSAT (*Algorithm* 1) for a state x and an exemplary 3-SAT instance, where a literal associated with variable $x_{k}$ (${\overset{¯}{x}}_{k}$) is denoted by k (−k). The number of critical clauses broken by flipping a variable $x_{k}$ is represented by $b_{k}$, and the change in the TLC by $T_{k}$. The greedy step in WalkSAT flips the variable with minimal b. Instead DOC amounts to introducing the minimization of T, which leaves the fewest number of true literals after the flip. DOCSAT uses a weighted compromise of both incentives by minimizing a weighted score (Eq. **4**). The central schematic illustrates the average effect of the individual processes on the true literal patterns: WalkSAT avoids breaking clauses, on average leading to more oversatisfied clauses, while DOC has the opposite effect (cf. Fig. 1 *Upper* panel).

**Fig. 4.**
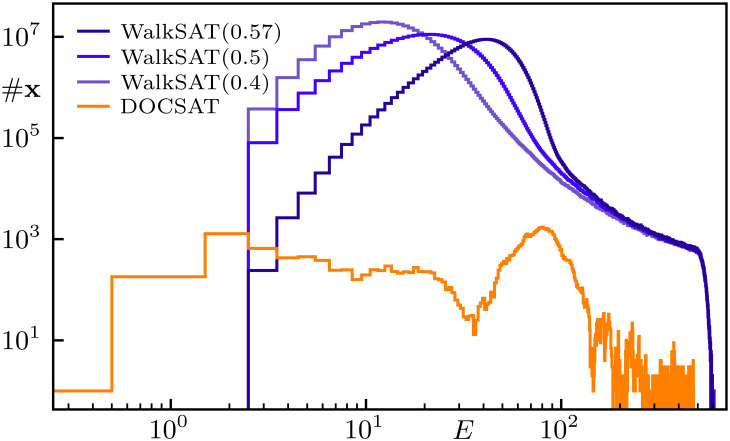
Logarithmic plot of the number of states #x with energy E for a hard 3-SAT instance with N=1,000 at $\alpha =\alpha_{\text{crit}}$ over $10^{3}$ trials with $3\cdot 10^{5}$ flips each. The walk probabilities $P_{\text{walk}}$ for WalkSAT are displayed in the legend; DOCSAT uses $P_{\text{walk}}=0.4$ and $r_{\text{doc}}=0.15$. Note that the solution (E=0) is at the left of the plot and that DOCSAT terminates after finding it, thus not exhausting all iterations.

**Fig. 5.**
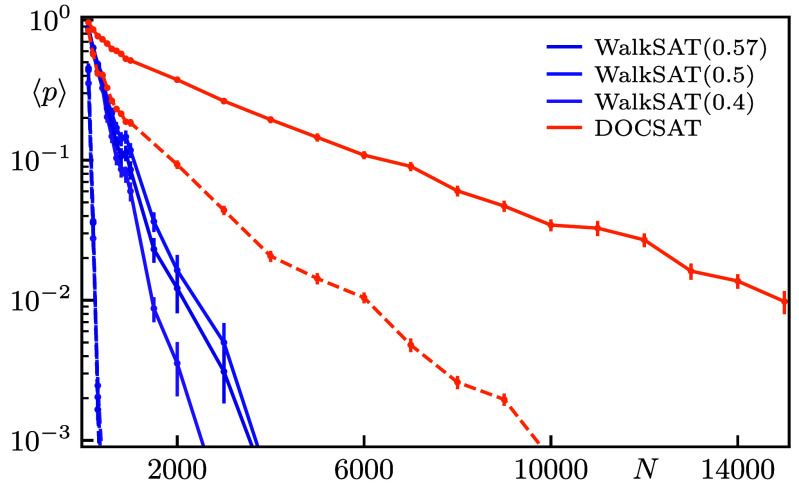
Average success probability ⟨p⟩ for the same dataset as in Fig. 1. The walk probabilities $P_{\text{walk}}$ for WalkSAT are displayed in the legend; DOCSAT uses $P_{\text{walk}}=0.4$ and $r_{\text{doc}}=0.15$. For the dashed lines, the average is taken over the hardest quintile (50 instances) for each solver. Errors correspond to the SD of the mean.

Hard combinatorial problems such as the Nondeterministic Polynomial Time (NP) complete satisfiability problem 3-SAT (1–3) are of ubiquitous importance, both from a scientific perspective and for a wide range of applications from integrated circuit design (4, 5), model checking (6, 7) and logistics (8–10) via frustrated magnetism (11, 12) to protein folding (13, 14) and AI development (15–18). Although a full solution to such universal problems remains elusive and is not even expected to become viable with the advent of quantum computers (19, 20), their gravitas has motivated intense research efforts for decades. Besides conceptual advances in complexity theory (20), impressive progress toward unlocking more and more complex 3-SAT problem instances has been made in at least two directions (6, 21–24). First, the practical performance of so-called complete solvers proven to eventually master any instance, albeit at the expense of exponentially long worst-case runtime, has tremendously improved (25, 26). Second, stochastic solvers such as WalkSAT that heuristically approach the problem by sampling techniques frequently solve even hard instances up to quite remarkable problem sizes (27–29). Further improving on this latter class of stochastic computational tools is the main objective of our present article. Below, we introduce and benchmark a stochastic 3-SAT solver coined DOCSAT (cf. *Algorithm* 1) which provides a major advance in solving critically hard instances as compared to existing algorithms (Fig. 1). The underlying heuristic is based on the crucial insight that the landscape of local and global minima has additional structure beyond the natural 3-SAT cost function (energy) that simply counts unsatisfied constraints. Specifically, we find that local minima in which WalkSAT is prone to get trapped for hard instances are distinguished from global minima (true solutions) by containing significantly more oversatisfied constraints (Fig. 2), i.e. combinatorial expressions that would remain logically true upon changing one of their correct Boolean variables (cf. Fig. 1*Upper* panel). Building on the celebrated WalkSAT heuristic (30), our DOCSAT solver (see Fig. 1*Upper* panel and Fig. 3 for illustration) exploits this structure by dissipating oversatisfied constraints (DOC), thus rendering more constraints critically satisfied so as to escape the aforementioned local minima toward true solutions of hard instances (cf. Fig. 4). Considering the hardest quintile from our sample of Weigt-type benchmark instances (31) in the critical regime, we demonstrate that DOCSAT performs stronger even on this unfavorable selection than WalkSAT does on the entire sample (Fig. 5).

**Fig. 6.**
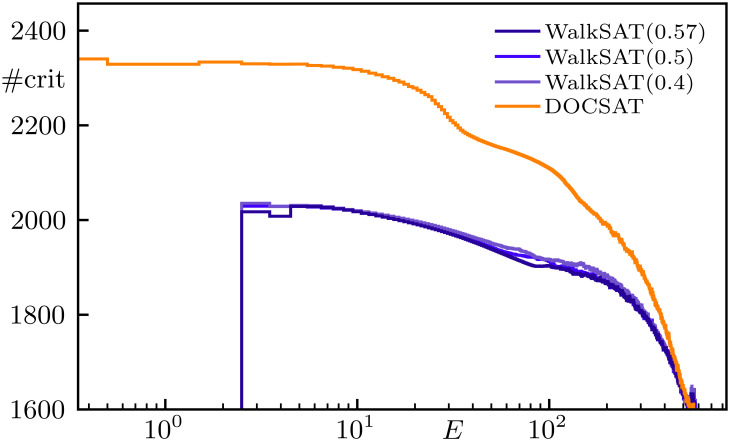
Average number of critical clauses #crit in states with energy E for the hard N=1,000 instance from Fig. 4 over 100 trials with $3\cdot 10^{5}$ flips each. The walk probabilities $P_{\text{walk}}$ for WalkSAT are displayed in the legend; DOCSAT uses $P_{\text{walk}}=0.4$ and $r_{\text{doc}}=0.15$.

**Fig. 7.**
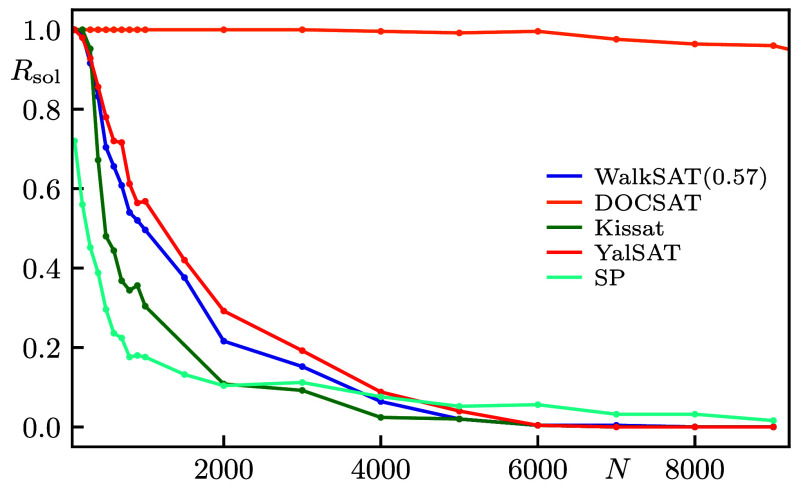
Fraction $R_{\text{sol}}$ of solved instances for algorithms YalSAT, Kissat, and SP for the same benchmark instances as in Fig. 1. For comparison, we also repeat the best scaling WalkSAT with $P_{\text{walk}}=0.57$ and DOCSAT with $P_{\text{walk}}=0.4$ and $r_{\text{doc}}=0.15$.

**Fig. 8.**
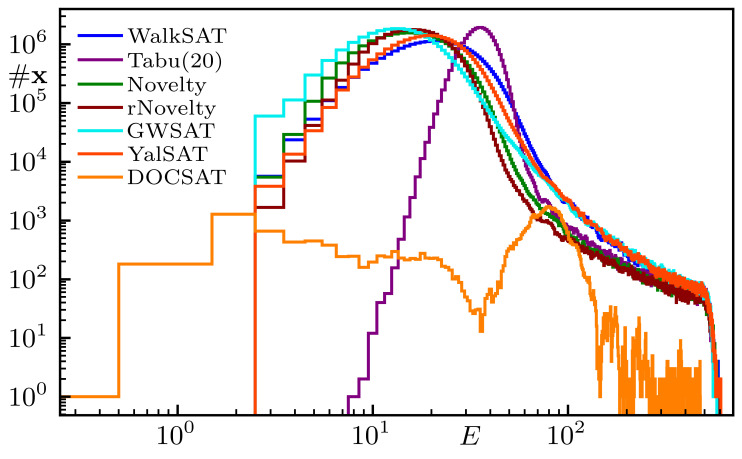
WalkSAT and related SLS algorithms for a hard N=1,000 instance readily solved by DOCSAT (cf. Fig. 4). The walk probability is fixed to $P_{\text{walk}}=0.5$; see the main text for further information on the displayed solvers and references.

We also verify with benchmark data that the DOCSAT heuristic indeed generates critical clauses at the expense of oversatisfied clauses at a higher rate than WalkSAT (Fig. 6). Finally, to broaden the scope of our benchmark, we compare DOCSAT to a wide range of previous algorithms, including both Stochastic local search (SLS) and complete solvers (Figs. 7 and 8).

## Results

### Structure Behind Low-Energy Caveats of WalkSAT.

The motivation for our DOCSAT solver (cf. next section) is based on insights regarding the landscape of local minima that limit the performance of existing stochastic algorithms such as WalkSAT, with particular reference to hard critical 3-SAT instances generated by the Weigt-protocol (31) (cf. *Algorithm* 2). To establish notation, we start by briefly discussing the key ingredients of our analysis, i.e. the 3-SAT problem and the WalkSAT heuristic.

A 3-SAT problem consists of N Boolean variables $x=(x_{1},\cdots ,x_{N})$ constrained by M=αN clauses, with the clause density α. Every clause $C_{m}$ consists of three literals, $C_{m}=l_{m,1}\vee l_{m,2}\vee l_{m,3}$, that can be either a variable (positive literal, e.g. $l_{m,1}=x_{m_{1}}$) or its negation (negative literal, e.g. $l_{m,1}={\overset{¯}{x}}_{m_{1}}$). An assignment (or bitstring) x solves the problem if all the clauses $C=(C_{1},\cdots ,C_{M})$ simultaneously evaluate to true under it. Formally, a 3-SAT problem is a Boolean formula in conjunctive normal form (CNF), i.e. $C_{1}\wedge \ldots \wedge C_{M}$. For any assignment x, we define its energy as

[1] $$\begin{array}{cc} E(x) & =\text{number of clauses violated by}\;x, \end{array}$$

such that solutions are distinguished by E=0, while each unsatisfied constraint contributes an energy penalty of +1. As an example, the clause $C_{1}=x_{1}\vee {\overset{¯}{x}}_{3}\vee {\overset{¯}{x}}_{7}$ is satisfied by 7 out of the $2^{3}=8$ possible assignments to the involved triple of variables. Note that under insertion of an assignment, say $x_{1}=1$, $x_{3}=0$, and $x_{7}=1$, the individual literals of the clause $C_{1}$ become either true ($x_{1}=1$, ${\overset{¯}{x}}_{3}=1$) or false (${\overset{¯}{x}}_{7}=0$). We define the TLC of an assignment x as

[2] $$\begin{array}{cc} \text{TLC}(x) & =\text{total number of true literals in C}. \end{array}$$

For the explicit example above, the clause $C_{1}$ contributes 2 true literals to TLC(x), because both $x_{1}$ and ${\overset{¯}{x}}_{3}$ evaluate to true under the assignment in the example. Note that every other occurrence of $x_{1}$ or ${\overset{¯}{x}}_{3}$ in subsequent clauses would further increase the count. For a satisfying assignment x, every clause must contain at least one true literal. We refer to clauses with more than one true literal as oversatisfied, as opposed to critical clauses (one true literal) and unsatisfied clauses (no true literal), respectively (cf. Fig. 1*Upper* panel). Promoting critical clauses at the expense of oversatisfied clauses is at the heart of our present work.

SLS solvers approach a 3-SAT problem via a focused search starting from a random initial state x (27–30). Focused means that every iteration of the algorithm starts by (randomly) selecting one of the unsatisfied clauses. Guided by a heuristic, one then flips a variable associated with a literal in this clause, satisfying it in the process. This is iterated for a fixed number of flips, and then the search can be restarted from a new random state. The essence of such SLS algorithms then lies in the heuristic for selecting variables and clauses.

In the SLS heuristic WalkSAT, one keeps track of the breakcount b for every variable, which is the number of clauses that would be broken by flipping that variable. Then, with a certain probability $\left(1-p_{\text{walk}}\right)$, one performs a greedy step by picking a variable with minimal breakcount in a randomly chosen unsatisfied clause. With the remaining walk probability $P_{\text{walk}}$, one instead mimics a random walk by flipping a variable at random within the aforementioned unsatisfied clause; this noise provides the major advantage over the GSAT (Greedy SAT) heuristic (30). Finally, if a breakcount is zero, that variable is always selected (i.e. “free” moves are preferred). Note that WalkSAT can be recovered from our DOCSAT heuristic (cf. *Algorithm* 1; see Fig. 3 for an illustration) by setting the parameter $r_{\text{doc}}=0$ in Eq. **4**. A WalkSAT implementation can be found in ref. 32, which is the version we adapted for our benchmarks.

#### True literal counting statistics.

We are now ready to reveal the crucial structure of low-energy minima that motivates our DOCSAT heuristic. Using the Weigt-protocol (31), we generate 250 problem instances with N=200 variables with critical clause density $\alpha_{\text{crit}}=4.27$, which corresponds to the hardest regime of 3-SAT at a given N (31, 33, 34). For each instance, we run WalkSAT for $10^{4}$ trials with $10^{4}$ flips per trial with walk probability $P_{\text{walk}}=0.5$. To study the low-energy landscape, we record the energy E and TLC, i.e. the total number of true literals, of every assignment occurring during the search.

Generally speaking, WalkSAT shows strong variability in the success probability $P=\frac{\text{successful trials}}{\text{total trials}}$, and we refer to the extremes as (comparably) hard (P<1%) and easy (P>90%) instances, thus yielding 29 hard and 26 easy instances. Remarkably, as shown in Fig. 2, we observe that the discrepancy between TLC(E=0) and TLC(E>0) is clearly correlated with P: For easy problems, the distribution of solution states (E=0) has TLCs similar to that of the low-energy sector (exemplified by E=5). By contrast, for the hard problems, all of the (few) solutions found have significantly lower TLC. This quantitatively substantiates the quite natural picture that solutions to hard instances should be fragile in the sense of combining many critical constraints. In summary, we conclude that WalkSAT is prone to ending up in a regime of high TLC, a desert of low-energy minima that is far from a true solution.

### The Dissipating Oversatisfied Constraints Heuristic.

Having identified a high TLC as an obstruction to WalkSAT, we now introduce a heuristic aimed at addressing this issue. To start with, note that finding a state with minimal or maximal number of true literals is easy, because the problem factorizes into one for each variable: To determine whether say $x_{3}$ should be set to true or false in the interest of TLC, one merely needs to count the number of positive and negative occurrences of $x_{3}$ in the clauses, which can be stored in N-vectors p and n respectively. Note that p and n are fixed for a problem instance and do not depend on the assignment x. A positive difference ${\left(p-n\right)}_{3}$ then implies that $x_{3}=1$ in the assignment with maximal TLC and $x_{3}=0$ in the one with minimal TLC.

Our goal is to reduce the TLC by DOC. Instead of minimizing the breakcount b as in WalkSAT, we can define a pure DOC-heuristic that minimizes the TLC, leaving as few true literals as possible after a flip. For a literal l associated with the k-th Boolean variable, the change in the TLC for flipping $x_{k}$ is given by

[3] $$\begin{array}{c} T={\left(p-n\right)}_{k}\cdot \left(2x_{k}-1\right). \end{array}$$

In Fig. 3, we illustrate the individual optimization with respect to b and T for a concrete example. Both figures of merit have their advantages and caveats. b tends to reduce energy but accumulates oversatisfied constraints driving WalkSAT into the aforementioned local-minima desert. T is designed to reduce oversatisfied constraints at the expense of occasionally increasing energy, thus the terminology DOC. As a trade-off aimed at combining the benefits of both, we introduce a total score

[4] $$\begin{array}{cc} s & =b+r_{\text{doc}}\cdot T, \end{array}$$

where $r_{\text{doc}}$ is the relative weight of the DOC-term. This implements a pull toward lower TLC because the new term can act as a tiebreaker for identical breakcounts even for small $r_{\text{doc}}$, thus favoring critical clauses. The resulting pseudocode for selecting a variable to be flipped in a randomly chosen unsatisfied clause is given in *Algorithm* 1.

**Algorithm 1** Variable selection in DOCSAT

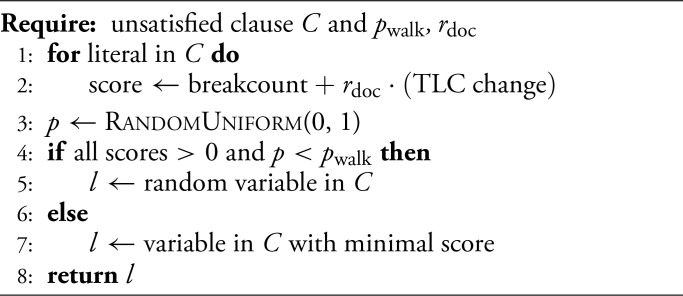

By viewing true literals as a finite resource, we can gain an intuitive understanding of the mechanism behind DOC. Consider having to choose between two states with identical (and low) energy E, but different TLC. Trying to fix the remaining broken clauses demands an increase in TLC by introducing new true literals, and because the TLC can not be increased indefinitely, the state with fewer true literals can be interpreted as the more efficient assignment. Additionally, from a probabilistic standpoint, the chances of introducing new true literals during the process of satisfying the remaining clauses are higher in that state: The lower the TLC is, the more likely a flip of a false literal is to produce true literals elsewhere. As such, the TLC amounts to a refinement of the search criterion for states beyond their energy.

Tying this back into our discussion at the end of the previous section, we expect DOC to be particularly useful when the solution states are fragile in the sense of containing many critically satisfied constraints, as appears to be common for the hardest Weigt-type instances.

### Performance Benchmark.

In WalkSAT, it is known empirically that $P_{\text{walk}}\approx 0.57$ is optimal for large random 3-SAT instances (35, 36). To demonstrate that fine-tuning this parameter does not significantly influence the performance, we also show variants with $P_{\text{walk}}=0.4$—which proves superior in some smaller examples—and $P_{\text{walk}}=0.5$. For DOCSAT, we choose $P_{\text{walk}}=0.4$ and $r_{\text{doc}}=0.15$.

With the DOCSAT heuristic, we are able to solve all of the N=200 instances from our previous benchmark (cf. Fig. 2). The potential of the algorithm is further demonstrated in Fig. 4, where we visualize the states found by WalkSAT and DOCSAT for a hard instance with N=1,000. There, we show the number of states #x found as a function of energy E over $10^{3}$ trials with $3\cdot 10^{5}$ flips each; DOCSAT is terminated after finding the solution, as it would just oscillate between E=0 and E≥1 afterward. Note that WalkSAT gets stuck at E=3 and that this instance also appears out of reach for current state-of-the-art complete solvers such as Kissat (37, 38). More generally, we stress that for the hard critical instances studied in our present work, more complex algorithms do not (significantly) outperform WalkSAT, which, apart from simplicity, motivates our focus on benchmarking our findings against WalkSAT in the following. In *Comparison to Further Algorithms*, we provide further benchmark data for other algorithms and variants.

For a more in-depth benchmark, we analyze samples of 250 instances each for problem sizes from N=100 to N=15,000 at the critical clause density $\alpha_{\text{crit}}=4.27$. For WalkSAT and DOCSAT, we run $10^{3}$ trials with 300N flips per trial, where the linear scaling in system size is to avoid an artificial decrease in success probability. In Fig. 1, we visualize the solution ratio $R_{\text{sol}}=\frac{\text{solved instances}}{\text{total instances}}$ as a function of N, demonstrating the reliably strong performance of DOCSAT in the critically hard realm for unprecedented system sizes.

As a different representation of the benchmark dataset, we also show the ensemble-averaged success probability per run ⟨p⟩ in Fig. 5, where the expected runtime for finding a solution scales as 1/⟨p⟩. Additionally, the figure contains the average over the hardest quintile of instances for each solver, which filters out (comparably) easy problems.

The data are consistent with an exponential fit

[5] $$\begin{array}{c} \left\langle p\right\rangle ∼{\left(1+b\right)}^{-N}, \end{array}$$

in agreement with an asymptotically exponential runtime. Quite remarkably, the asymptotic scaling of DOCSAT is found to be substantially superior to WalkSAT in all aspects. Specifically, in Eq. **5** for the entire benchmark sample, we find $b\approx 2\cdot 10^{-3}$ for the best-scaling WalkSAT parameter $P_{\text{walk}}=0.57$, while $b\approx 3\cdot 10^{-4}$ for DOCSAT. For the hardest quintile, we instead find $b\approx 3\cdot 10^{-2}$ for WalkSAT, and $b\approx 6\cdot 10^{-4}$ for DOCSAT, respectively. The latter observation does not only demonstrate that DOCSAT performs stronger on the hardest quintile than WalkSAT does on average but also that the relative loss of performance when comparing the entire sample with the hardest quintile is much smaller for DOCSAT.

Finally, we find it interesting to analyze the generation of critical clauses at the expense of oversatisfied ones in DOCSAT, which was the main intuition behind its construction. Upon selecting and flipping a variable in an unsatisfied clause, all the other clauses in which the variable appears change too. Indeed, the rate $\Gamma_{c}$ at which critical clauses are increased by the nonrandom step (see line 8 in *Algorithm* 1) for DOCSAT is about a factor of 4 higher than for WalkSAT ($r_{\text{doc}}=0$ in *Algorithm* 1). Also including the transitions from unsatisfied (in addition to those from oversatisfied) to critical clauses, the overall generation of critical clauses in DOCSAT remains larger by a factor of roughly 2. To illustrate this characteristic behavior in greater depth, in Fig. 6, we compare WalkSAT and DOCSAT regarding the average number of critical clauses in assignments occurring during the (attempted) solution of a hard 3-SAT instance of size N=1,000 (cf. Fig. 4) as a function of energy. Clearly, DOCSAT maintains a higher number of critical clauses throughout its search, and the difference to WalkSAT increases with decreasing energy, which we deem crucial to DOCSAT’s capability of actually finding the global minimum (E=0).

These properties establish a specific relation between the guiding principle behind the construction of the DOCSAT heuristic and the empirical observation of its practical performance.

### Comparison to Further Algorithms.

In this section, we illustrate the performance of two other SAT solvers, namely the SLS solver YalSAT (winner of the uniform-random track in 2017) (39), which builds on ProbSAT (36), and the complete solver Kissat (winner in all main tracks in 2024) (37, 38). We also include Survey Propagation (SP), which is the best known algorithm for finding solutions to very large random uniform problems close to $\alpha_{\text{crit}}$ (40, 41). Even at critical clause density, problems with N=200 are still sufficiently easy for Kissat (YalSAT solves all but a few of the instances that are hardest for WalkSAT). However, although one can often solve practical problems with millions of variables in many applications, critically hard random instances—such as the Weigt-protocol instances studied in our present benchmark—already become challenging at much smaller N even for these most advanced solvers (Fig. 7). Also SP does not work well on our benchmark sample. In many cases, already a few variable fixes render the problem unsatisfiable (at small N provably so; sometimes after the very first variable). We therefore conclude that the statistical biases exploited by SP in random uniform instances are absent in the majority of Weigt instances, further emphasizing their lack of such structure.

Due to the significantly more complex nature of these solvers, we refer to the respective original publications for a detailed description. Both YalSAT and Kissat are run with the default settings used for the competitions (except hinting Kissat at targeting satisfiable instances). YalSAT follows the usual SLS structure of using heuristics to select clauses and variables, so we set the same limit of 300N flips per trial as for WalkSAT and DOCSAT. Because the heuristics are more involved, this results in a slightly longer runtime granted to YalSAT. For Kissat, we instead use a cutoff on the maximal number of so-called decisions during the search. While this has the advantage of being a hardware-independent measure, we note that the runtime scales slightly superlinear in the decisions. Since the solver is complete we only run it once with at least comparable total walltime to compensate for the $10^{3}$ restarts of the SLS solvers. Specifically, using 3,000N decisions in Kissat yields similar walltime as YalSAT at N=500 with an additional factor of up to 2 in favor of Kissat toward N=9,000. For the voting phase in SP, we use $10^{3}$ iterations with tolerance $\epsilon =10^{-3}$ and 1% of variables fixed per cycle [cf. (40)]. SP itself benefits little from restarts, i.e. the success probability P is usually close to 0 or 1 for a given problem. Thus, we only run SP 20 times, but with 50 WalkSAT trials per run for comparable overall runtime. Note that at moderate N, SP still performs worse than plain WalkSAT despite the same $10^{3}$ total trials, which is consistent with our observation that the voting phase often makes Weigt instances unsatisfiable. In Fig. 7, we compare the fraction of solved instances for all aforementioned solvers with DOCSAT, further corroborating its superior performance (cf. Fig. 1).

In Fig. 8, we benchmark YalSAT on the hard N=1,000 instance used for Fig. 4. There we also include some of the (relatively minor) variations on WalkSAT such as Novelty and GWSAT (42, 43). Tabu is WalkSAT with a list of recently flipped variables (here 20) that are not allowed to be flipped back; this avoids loops, but evidently is not very helpful for these random instances. GWSAT takes into account how many unsatisfied clauses are fixed by flipping a variable (so-called makecount m) and optimizes b−m instead of just b (corresponds to the greedy algorithm GSAT combined with random walks). Curiously, even YalSAT follows a very similar curve, while the behavior of DOCSAT is drastically different and converges to the full solution well before exhausting the iteration limit (cf. Fig. 4).

## Concluding Discussion

We have introduced a SLS heuristic coined DOCSAT that significantly improves on existing stochastic 3-SAT solvers in the realm of particularly hard satisfiable instances by reducing the number of oversatisfied combinatorial constraints so as to promote critical clauses. Our construction is based on the analysis of states found by WalkSAT in the low-energy regime for satisfiable 3-SAT problems at the notoriously difficult critical value of the clause density, specifically on our observation that solutions typically have fewer true literals than readily found local minima. As our numerical simulations demonstrate, exploiting this statistical property yields substantial improvement over both WalkSAT and other modern solvers.

Adopting a broader perspective, the idea of DOC can be interpreted as identifying a criterion to guide the SLS toward more favorable states that is complementary to the number of violations (energy), i.e. the primary cost function of 3-SAT. We note that for natural physical problems, additional structure such as symmetries and spatially local correlations commonly distinguish beyond the physical notion of energy ground states from say random states. In this sense, inspired by the search for such additional structure, our implementation of DOC provides a minimal working example for escaping a vast landscape of local minima characterized by a certain statistical property distinguishing true solutions in the context of abstract combinatorial problems. In particular, moving beyond true literal counts, one could also generalize this concept to distributions of true literal patterns or directly target already satisfied constraints.

## Materials and Methods

Solver methods are described in the main text and algorithms for numerical benchmarks are implemented in Python and C without any nonstandard libraries. For generating hard random but satisfiable 3-SAT problem instances, we use the Weigt-protocol (31), which we reproduce in the form of *Algorithm* 2 for the readers convenience.

**Algorithm 2** Generating hard satisfiable random instances

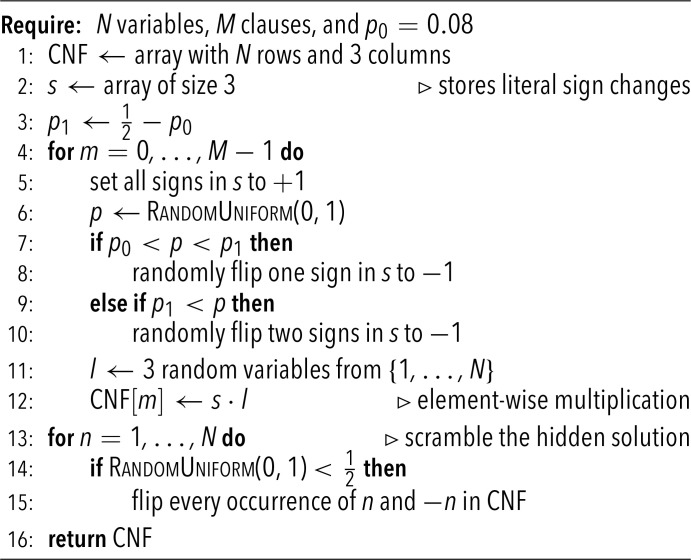

Particularly hard instances are expected for $p_{0}\in \left(0.077,0.25\right)$, and we set $p_{0}=0.08$ in our main benchmark. The motivation for this choice is that smaller values of $p_{0}$ are expected to lead to a more even distribution of clause types. In particular, clauses with exactly two true literals in the planted solution vanish when $p_{0}\to 0.25$; small $p_{0}$ instead yield distributions closer to fully random problems, which should mitigate unintentional structure.

## Data Availability

All study data are included in the main text.
